# Epidemiology of Thyroid Cancer in Tuscany (Central Italy) 2013–2017: Not Just Overdiagnosis

**DOI:** 10.3390/cancers17050717

**Published:** 2025-02-20

**Authors:** Marco Capezzone, Liborio Torregrossa, Anello Marcello Poma, Alessandra Cartocci, Luisa Petrone, Clotilde Sparano, Matteo Puccioni, Daniele Barbaro, Daniela Bigini, Annamaria Marcantonio, Cinzia Pupilli, Cristina Ladu, Elena Gianetti, Chiara Vezzosi, Valentina Belardini, Virginia Mancini, Eugenia Maria Morabito, Gilda Dalmazio, Massimo Tosti Balducci, Nicola Libertà Decarli, Giacomo Giubbolini, Simone Boccuzzi, Massimo Alessandri, Paolo Piacentini, Caterina Di Cosmo, Melissa De Servi, Luca Tomisti, Marco Pellegri, Giovanni Gravina, Marco Ceroti, Luigi De Napoli, Gabriele Materazzi

**Affiliations:** 1 UOSD of Endocrinology, Misericordia Hospital, Senese Street 146, 58100 Grosseto, Italy; eugeniamaria.morabito@uslsudest.toscana.it (E.M.M.); gilda.dalmazio@uslsudest.toscana.it (G.D.); massimo.tostibalducci@uslsudest.toscana.it (M.T.B.); massimo2.alessandri@uslsudest.toscana.it (M.A.); 2 Department of Surgical, Medical, Molecular Pathology and Critical Area, University of Pisa, 56124 Pisa, Italy; liborio.torregrossa@unipi.it (L.T.); marcello.poma@med.unipi.it (A.M.P.); 3 Department of Medical Biotechnologies Bioengineering Lab, University of Siena, 53100 Siena, Italy; alessandra.cartocci@dbm.unisi.it; 4 Endocrinology Unit, Department of Experimental and Clinical Biomedical Sciences “Mario Serio”, University of Florence, 50134 Florence, Italy; luisa.petrone@unifi.it (L.P.); clotilde.sparano@unifi.it (C.S.); matteo.puccioni@unifi.it (M.P.); 5 U.O.S.D. Endocrinology in Livorno Hospital, USL Nordovest Toscana, 57100 Livorno, Italy; 6 UOSD Diagnostic Cytology ASL North West, 55049 Tuscany, Italy; daniela.bigini@uslnordovest.toscana.it; 7 SOSD Endocrinology ASL Center, 50125 Tuscany, Italy; annamaria.marcantonio@uslcentro.toscana.it (A.M.); cinzia.pupilli@uslcentro.toscana.it (C.P.); cristina.ladu@uslcentro.toscana.it (C.L.); elena.gianetti@uslcentr.toscana.it (E.G.); 8 Endocrinology Unit, San Donato Hospital, 52100 Arezzo, Italy; chiara.vezzosi@uslsudest.toscana.it (C.V.); valentina.belardini@uslsudest.toscana.it (V.B.); 9 Section of Pathology, Department of Medical Biotechnology, University of Siena, 53100 Siena, Italy; 10 Department of Pathology, Misericordia Hospital, 58100 Grosseto, Italy; nicolaliberta.decarli@uslsudest.toscana.it (N.L.D.); giacomo.giubbolini@uslsudest.toscana.it (G.G.); 11 Department of Otorhinolaryngology, Misericordia Hospital, 58100 Grosseto, Italy; simone.boccuzzi@uslsudest.toscana.it; 12 Unit of Epidemiology, Department of Prevention Misericordia Hospital, 58100 Grosseto, Italy; paolo.piacentini@uslsudest.toscana.it; 13 UOSD of Endocrinology, Versilia Hospital, 55041 Lido di Camaiore, Italy; caterina.dicosmo@uslnordovest.toscana.it; 14 Endocrinology Unit, Massa-Carrara Hospital ASL North West, 54100 Tuscany, Italy; melissa.deservi@uslnordovest.toscana.it (M.D.S.); luca.tomisti@uslnordovest.toscana.it (L.T.); 15 Nuclear Medicine Unit, San Luca Hospital, 55100 Lucca, Italy; marco.pellegri@uslnordovest.toscana.it; 16 Endocrinology Section, Department of Medicine San Rossore Clinic, 56122 Pisa, Italy; gravina@sanrossorecura.it; 17 Tuscany Cancer Registry, Clinical and Descriptive Epidemiology Unit, Institute for Cancer Research Prevention and Clinical Network (ISPRO), 50139 Florence, Italy; m.ceroti@ispro.toscana.it; 18 Division of Endocrine Surgery, Department of Surgical Pathology, University Hospital of Pisa, 56124 Pisa, Italy; l.denapoli@ao-pisa.toscana.it (L.D.N.); gabriele.materazzi@unipi.it (G.M.)

**Keywords:** epidemiology, incidence, thyroid carcinoma, heavy metals, pollution

## Abstract

Background: Thyroid cancer (TC) incidence, particularly the papillary histotype (PTC), has been increasing worldwide, and some of the highest incidence rates (IRs) have been reported in Italy, although with intra-country heterogeneity. The increasing incidence of TC is predominantly due to overdiagnosis, but environmental and lifestyle causative factors cannot be excluded. The aim of this study was to report TC incidence and mortality rates in the Tuscany region (Central Italy) and to review the histological records of TC patients born and living in the region, according to the province of residence, particularly in municipalities where heavy metal pollution has been well documented. Methods: The Tuscany Cancer Registry (ISPRO), certified by the Italian Association of Cancer Registries (AIRTUM), provided us with the number of cases and European age-standardized IRs of TC patients for all 10 Tuscany provinces from 1 January 2013 through 31 December 2017. In addition, we collected the TC histological records and diagnosed them in the same period. Results: A total of 4459 cases, 3209 (72%) women and 1250 (28%) men, were diagnosed with TC and reported by ISPRO. In women, the age-standardized IRs ranged from 22.3 to 45.4 cases per 100,000 inhabitants; in men, this ranged from 8.4 to 16.5 cases per 100,000 inhabitants. The histological records of TC patients living in the Tuscany provinces of the coastal areas (Livorno, Grosseto, Pisa, Massa-Carrara, and Lucca) appeared phenotypically more aggressive (higher extrathyroidal invasion, higher rate of lymph node metastases, higher rate of tumor bilaterality and multicentricity, lower rate of microcarcinomas) compared to those of the other provinces. When evaluating the relative risk (RR) of diagnosing TC in relation to environmental sources of heavy metal pollution exposure, we found that exposed patients had an RR of 1.16 (95% CI: 1.04–1.29), significantly greater compared to non-exposed patients. Conclusions: The overdiagnosis phenomenon is the main explanation for the increased incidence of TC in Tuscany. However, in some geographical areas of the region, the presence of environmental pollution, especially that characterized by the release of heavy metals, might influence TC incidence rates and the phenotype.

## Simple Summary

In recent decades, thyroid cancer (TC) incidence has increased worldwide, and the overdiagnosis phenomenon has been reported to represent the main explanation. However, there is growing evidence to support a true rise in thyroid cancer incidence, and the impact of common environmental exposures remains understudied. We reported the incidence rates of TC in the Tuscany region (central Italy) from 1 January 2013 through 31 December 2017 and the correlation with histological records of TC patients. In addition, we evaluated the relative risk of diagnosing TC in relation to exposure to local environmental sources of heavy metal pollution. The histological records of patients born and living in the coastal area of Tuscany appear phenotypically more aggressive compared to those of the other Tuscany provinces. We found that exposed patients to heavy metal pollution had a relative risk of diagnosing thyroid cancer significantly greater compared to non-exposed patients.

## 1. Introduction

Thyroid cancer (TC) is the most common endocrine malignancy, and its incidence continues to rise worldwide [1,2]. On the contrary, TC mortality rates have changed minimally over the past five decades [3,4]. Many authors have primarily attributed the increasing incidence of TC to the overdiagnosis phenomenon, defined as the diagnosis of a condition that would not have caused harm to the individual over their lifetime if left undetected [5]. These are mainly small, differentiated carcinomas of ≤1 cm, often discovered accidentally, that will remain asymptomatic. However, the overdiagnosis hypothesis does not explain why other human cancers with increased scrutiny have not followed the same trend of TC [6]. In addition, some population-based studies documented a significant increase in TC of all sizes and stages [7]. These observations may suggest that the increased incidence of TC, at least in some geographical regions, is real and not an apparent phenomenon [8]. The incidence of Italian TC is one of the highest in Europe, although it has intra-country heterogeneity [9]. One study reported that in Italy, the incidence of TC has almost doubled between 1991–1995 and 2001–2005 [10]. A recent observational, cross-sectional analysis on the incidence of TC conducted in 15 European Union countries showed that in 2019, the highest age-standardized incidence rates were observed in Italy [11]. Another study reported that in 2020, the highest incidence of TC, evaluated in age groups 10–19 years, in the female population was recorded in Italy [12]. Recently, a population-based study showed that in Italy, the incidence rates of TC are largely due to overdiagnosis, although an upward trend was seen until the early 2010s, followed by a downward trend [13]. However, in the absence of a single national tumor registry in Italy, the difficulties of comparing findings from population-based and hospital-based cancer registries should be considered, and multiple risk factors for TC are conceivable [14]. Tuscany is the fifth largest Italian region, located in Central Italy, and presents an environment with many different characteristics, including volcanic areas, rural and urban zones, and industrial and nonindustrial areas. Compared to other Italian regions, Tuscany is the only Italian region where geothermal energy is present. In addition, for about 30 centuries, Tuscany has been one of the most important Italian mining regions, centered on the extraction of sulfides or sulfide-bearing ores [15]. A Cancer Registry has been active for the whole Tuscany region since 2013, and before that year, previous epidemiological studies on the TC incidence rate in Tuscany were related only to the Prato and Florence provinces [10,16,17]. Recently, one study reported the presence of a high incidence of TC in Southern Tuscany (Grosseto province), which may be influenced by the presence of environmental heavy metal pollution [18]. The aim of this study was to evaluate the incidence of TC in the entire Tuscany region from 1 January 2013 through 31 December 2017 and to correlate this information with age at diagnosis, gender, and histopathological features derived from histological records (tumor stage, histotype, size, multicentricity and bilaterality of the tumor) of TC patients born and living in this region, particularly in Tuscany municipalities with ascertained sources of heavy metal pollution, represented by the presence of geothermal power plants, mines, National Priority Contaminated Sites [NPCSs] and higher levels of radon concentrations.

## 2. Materials and Methods

### 2.1. Study Population

Tuscany Region, located in central Italy, is 22,985 km^2^ large and has a population of around 3,664,000 inhabitants [19]. The population is distributed in 10 provinces (Firenze, Arezzo, Grosseto, Livorno, Lucca, Massa-Carrara, Pisa, Pistoia, Prato, and Siena), including 272 municipality districts. Regional Authority organizes the sanitary system through several entities that cover the whole regional territory: three Local Health Care Agencies (South-East Area vasta with Arezzo, Grosseto, and Siena provinces, Center Area vasta with Firenze, Pistoia and Prato provinces and North-West Area vasta with Livorno, Lucca, Massa-Carrara and Pisa provinces) and four University Clinical Centers (Firenze Careggi, Firenze Meyer, Pisa and Siena) [20]. The Tuscany Regional Council n.1359 of 21 October 1996 formally recognized the Oncological Network, Prevention, and Research Institute (ISPRO) as the regional reference center for preventive Oncology. ISPRO is certified by the Italian Association of Cancer Registries (AIRTUM), which performs validation checks on completeness of coverage, accuracy, and interpretation to assure standard quality [21]. ISPRO provided us with the number of cases and European age-standardized incidence and mortality rates of TC patients for all ten Tuscany provinces available only for the period starting from 1 January 2013 to 31 December 2017.

**Figure 1:**
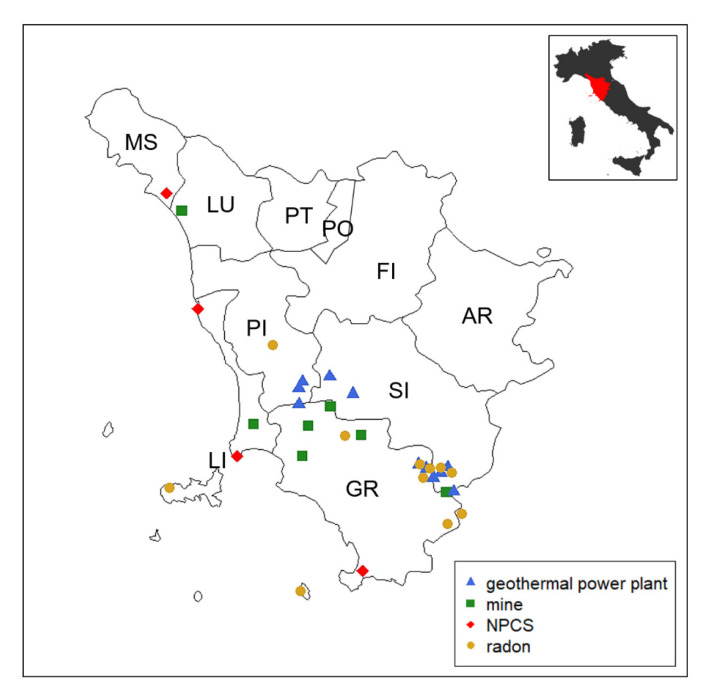
The map shows the Tuscany region, which is located in the central part of Italy. There are 10 provinces as highlighted in the map (AR, Arezzo; FI, Firenze; GR, Grosseto; LI, Livorno; LU, Lucca; MS, Massa-Carrara, PI, Pisa; PO, Prato; PT, Pistoia; SI, Siena). Municipalities exposed to GMNR (i.e., geothermal power plants, mines, national priority contaminated sites [NPCS], and radar) are marked on the map.

In addition, we examined the histological records of TC patients born and living in Tuscany who were diagnosed in the same period. Exclusion criteria were TC patients coming from other Italian regions or resident in Tuscany for less than 10 years and TC patients for whom it was not possible to verify the histological record. Histological records were collected from each provincial anatomical pathology center and reclassified according to the pTNM 8th edition criteria [22] by two independent endocrine pathologists. Clinical (age at diagnosis, sex, municipality of residence, type of surgery) and pathological data (tumor size, lymph node metastases, extrathyroidal extension, multifocality, and bilaterality of the tumor) were recorded in a database. The locations of principal mining areas in Tuscany are present in Metalliferous Hills (Grosseto province), in Campiglia and Elba Isle (Livorno province), in Santa Barbara Upper Valdarno lignite (Arezzo province), and in Mt. Amiata (Grosseto and Siena provinces). The mining maps are provided by the Tuscan Mining Geopark Archive while mining tunnels and galleries can be downloaded from the Data Bank Mineral Resources Tuscany Region [23]. NPCS management is entrusted to the Italian Ministry for the Ecological Transition—which uses the National Network System for Environmental Protection (SNPA, Rome, Italy) and the National Institute of Health (ISS, Rome, Italy), as well as other qualified public or private entities, for the technical investigation (Article 252-Legislative Decree 152/2006). To date, there are four NPCSs in Tuscany, and they are localized in Livorno (2 sites), Grosseto, and Massa-Carrara provinces [24]. In Italy, all of the geothermal fields in exploitation for electricity generation are located in Tuscany in Larderello Travale and Radicondoli (Pisa province), in Bagnore and Piancastagnaio (Mount Amiata area, Grosseto and Siena provinces) [25]. Areas with a high probability of high radon concentrations, a major source of ionizing radiation exposure for the general population, are considered those in which at least 10% of homes are estimated to exceed the reference level of 200 Bq/m^3^. There are 13 municipalities identified by the Environmental Protection Agency of Tuscany (ARPAT), with a total population of approximately 50,000 inhabitants (49,331 residents as of 31 December 2010, equal to approximately 1.3% of the regional total, ISTAT data) reported in the supplemental material (Supplemental Table S1) [26]. We used the acronym GMNR in the text to denominate the ascertained sources of heavy metal pollution considered in this study: Geothermal plants, mines, national priority contaminated sites, and radiation. The positions of GMNRs in the Tuscany region are reported in Figure 1.

### 2.2. Statistical Analysis

Continuous variables are presented as the median and interquartile range (IQR) and were tested by either the Mann–Whitney U test or Kruskal–Wallis’ test, followed by the Dunn’s test for pairwise comparisons. Associations between categorical variables were assessed by either the Chi-square test or Fisher’s exact test. For multiple contingency tables, the analysis of standardized residuals was performed to compute factor-level *p*-values. *p*-values were adjusted using the Bonferroni’s method. Incidence rate per 100,000 inhabitants, crude odd ratio (OR), and maximum likelihood 95% confidence intervals (CI) were computed following the procedures of the epiR R package v.2.0.75. The significance of OR was assessed by Fisher’s exact test. Average annual percent change (AAPC) and 95% CI were estimated after segmented regression and using the segmented R package v. 2.1-1. All analyses and graphics were produced in R environment (https://www.r-project.org/, v.4.4.1, last accessed 24 July 2024).

## 3. Results

### 3.1. Thyroid Cancer Incidence and Mortality Rates in Tuscany

**Figure 2:**
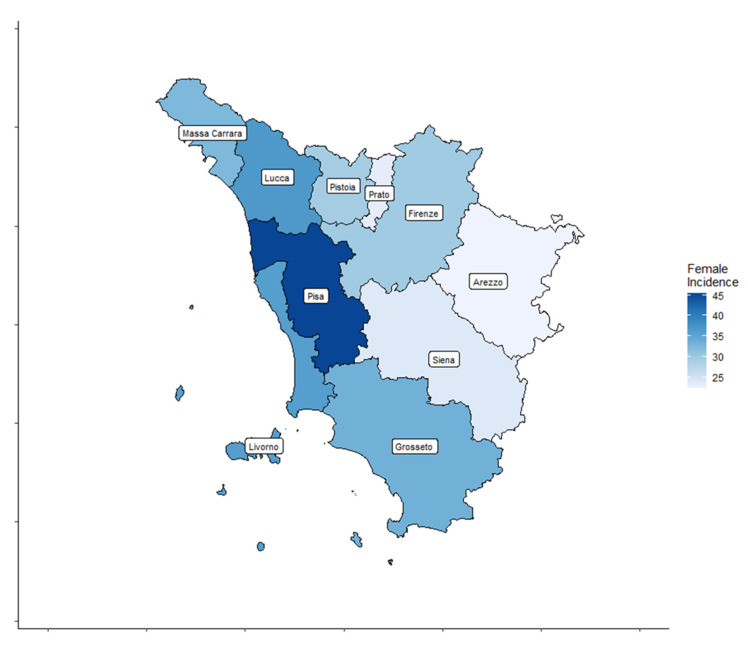
Thyroid cancer (TC) incidence rate distribution in the ten Tuscany provinces for female patients.

**Figure 3:**
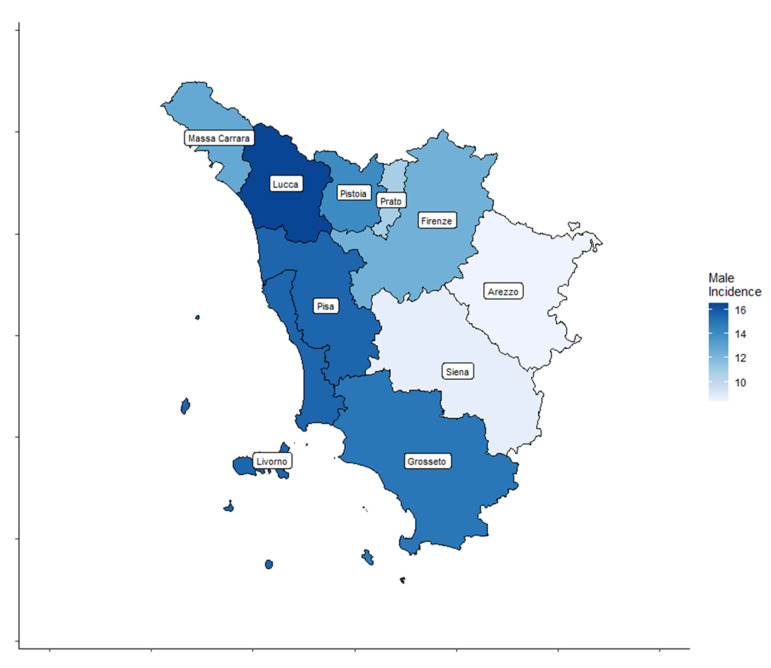
Thyroid cancer (TC) incidence rate distribution in the ten Tuscany provinces for male patients.

A total of 4459 cases, 3209 (72%) women and 1250 (28%) men, diagnosed with TC were registered by the ISPRO between 2013 and 2017. Age-standardized incidence rates for all thyroid cancer types are shown by Tuscany province (TP) for both sexes in Table 1 and Figure 2 and Figure 3.

In women, the age-standardized TC incidence rates ranged from 22.3 to 45.4 cases per 100,000 inhabitants. In men, the age-standardized TC incidence rates ranged from 8.4 to 16.5 cases per 100,000 inhabitants.

Age-standardized mortality rates for all thyroid cancer types in the corresponding period are shown by Tuscany province (TP) for both sexes in Table 2.

In women, the age-standardized TC mortality rates ranged from 0.47 to 0.91 cases per 100,000 inhabitants. In men, the age-standardized TC mortality rates ranged from 0.52 to 1.53 cases per 100,000 inhabitants.

### 3.2. Clinical and Pathological Features of TC Patients (n = 3210) Born and Living in Tuscany Region

We analyzed 3210/4459 (72%) histological records of TC patients born and living in the Tuscany region. The large majority of TC patients (3110/3210, 96.9%) underwent total thyroidectomy. Lymphadenectomy was performed in 509/3210 (15.8%) cases without significant differences between the ten Tuscany provinces. When thyroid cancers were analyzed by histotype, PTC was diagnosed in 2926 (91.1%) of the 3210 patients.

We found that 1339 (41.7%) of all thyroid tumors in Tuscany were microcarcinomas (maximum diameter ≤ 10 mm). The distribution of microcarcinomas was significantly different (*p* < 0.001) between the ten Tuscany provinces. In particular, a significantly lower rate was observed in Grosseto, Livorno, Massa, and Pisa compared to Firenze province. Similarly, the rate of extrathyroidal tumors was significantly higher (*p* < 0.001) in Grosseto, Livorno, Pisa, and Massa compared to Arezzo, Firenze, Lucca, Pistoia, and Siena. Moreover, the TC was significantly different between provinces in terms of multifocality (*p* = 0.001) and bilaterality (*p* < 0.001). In particular, patients who are residents of Grosseto, Livorno, and Pisa provinces had a higher prevalence of multifocal and bilateral TC compared to patients living in Arezzo, Firenze, Prato, Pistoia, and Lucca. In addition, patients living in the Pisa and Grosseto areas were significantly younger at diagnosis (*p* = 0.001) than patients living in Firenze and Prato. On the contrary, no statistically significant differences were observed regarding gender (*p* = 0.1) and the mean diameter of the tumor (*p* = 0.1) between the TPs (Table 3).

Clinical and Pathological Features of TC patients exposed (n = 385) and not exposed (n = 2825) to geothermal power plants, mines, NPCSs, and higher levels of radon concentrations (GMNRs). We identified 385 out of 3210 (12%) patients born and living in Tuscany municipalities characterized by the ascertained presence of environmental risk factors (GMNR-exposed patients). In exposed patients, the tumors were significantly characterized by papillary histotype (*p* = 0.03), multifocality (*p* = 0.002) and bilaterality (*p* < 0.001), higher prevalence of extrathyroidal invasion (*p* < 0.001), and a lower rate of microcarcinomas (*p* = 0.02) compared to GMNR-non-exposed patients. No statistically significant differences were found regarding age at diagnosis, sex, size of the tumor, and rate of lymph node metastases (Table 4).

### 3.3. Incidence Rate and Relative Risk of Thyroid Cancer in Relation to the Presence of GMNRs

**Figure 4:**
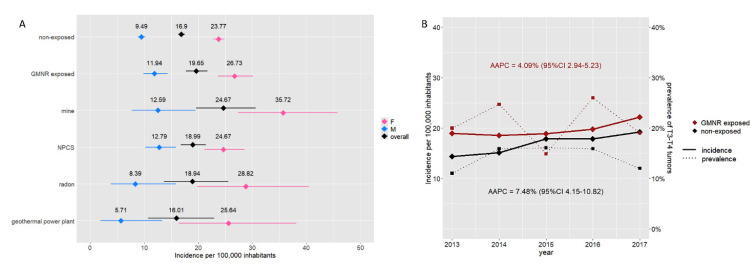
Incidence rate of thyroid cancer and GMNR exposition. Panel (**A**) Incidence rate per 100,000 inhabitants in the GMNR-exposed and GMNR-non-exposed in Tuscany. The graph shows the overall incidence (black) and incidence according to sex (i.e., blue for males and pink for females) in the GMNR-exposed and GMNR-non-exposed. Incidence rates according to the different pollution types are also reported. Horizontal lines represent 95% CI. Panel (**B**) Increment of incidence of thyroid cancer in both the GMNR-exposed (red) and GMNR-non-exposed (black) (AAPC 4.09% and 7.48%, respectively). No significant changes in the prevalence of T3–T4 tumors have been observed over the years (dotted lines).

We evaluated the relative risk of developing TC in relation to the presence of GMNRs. The crude incidence rate of TC in GMNR-exposed inhabitants was significantly higher than in the GMNR-non-exposed (19.65 vs. 16.90 per 100,000 inhabitants), with an RR of 1.16. The RR was greater in males than in females (1.26 and 1.12, respectively). As shown in Figure 4A, the greater incidence rate was observed in municipalities in the proximity of mines, while it was lower and comparable to the non-exposed in those near geothermal power plants. From 2013 to 2017, the incidence of TC increased in both the GMNR-exposed and GMNR-non-exposed, with an AAPC of 4.09% (95% CI 2.94–5.23) and 7.48% (95% CI 4.15–10.82), respectively. Despite the steady rise in the TC incidence rate over these years, no significant changes were observed in terms of the prevalence of T3 and T4 tumors (Figure 4B).

In NPCS-exposed people, a higher incidence was observed in males only (12.79 per 100,000 inhabitants, with an RR of 1.35); on the contrary, in municipalities with high levels of radon, a higher incidence of TC was observed in females only, though without reaching statistical significance (28.82 per 100,000 inhabitants, RR = 1.21). A detailed report of IRs and associated RR is reported in Table 5.

## 4. Discussion

Previous epidemiological studies, in the absence of a national cancer registry, reported TC incidence rates in the Tuscany region only for the Firenze and Prato provinces. Lise et al. reported that incidence rates (IRs) in women for both provinces Prato and Firenze ranged from 5.7/100,000 in the 1991–1995 period to 9.1 in the 2001–2005 period and IRs in men ranged in the same period from 2.0 to 3.7 [16]. Dal Maso et al. reported that in Firenze–Prato, the age-standardized incidence rates (ASR) in women increased from 14.9 in the 1998–2012 period to 27.5 in 2008–2012 and in men from 5.0 to 7.0 in the same period [17]. In both studies, the authors concluded that the heterogeneity of TC incidence rates was largely due to overdiagnosis and variations at a local level in medical surveillance. We observed that TC IRs (2013–2017 period) for the 10 TPs were extremely variable, confirming the situation previously reported in other Italian regions and mainly related to the overdiagnosis phenomenon. This hypothesis is strengthened by the observation that in some TPs, in particular Prato and Firenze, the rate of microcarcinomas was almost 50%. According to many other studies, the increased incidence of TC appeared to be restricted to the papillary histotype [27,28]. However, we observed that histological records of TC patients born and living in the coastal area of the region (Livorno, Grosseto, Pisa, Massa-Carrara, and Lucca) appear phenotypically more aggressive (higher extrathyroidal invasion, higher rate of lymph node metastases, higher rate of tumor bilaterality and multicentricity, lower rate of microcarcinomas) compared to those of the other TPs. The presence of potential bias, such as different therapeutic treatment or different access to healthcare institutions, is unlikely; all patients were born and/or live in Tuscany, where the public national health system is well-performing, and almost all TC patients were submitted to the same therapeutic approach (total thyroidectomy).

A number of modifiable risk factors have been identified over time to explain the increasing TC incidence in many countries. However, no evidence of increased exposure to known risk factors, at least not to the extent of explaining the steep increases and the large geographical heterogeneity in TC incidence, has been reported [29]. The only exception is environmental pollution, which increases climate change and represents the world’s largest environmental risk factor for disease, including cancer and premature death [30,31].

The typical heavy metal pollutants produced through urbanization, industrialization, and agricultural practices are heavy metals, and collectively, these metals represent a profound environmental risk factor for the development of several malignancies with some metals such as As, Cd, Cr, and Ni that are category-1 heavy metals according to the International Agency for Research on Cancer (IARC) [32]. The thyroid gland may be specifically affected, compared to other organs, because of its biological characteristics, and some carcinogenic metals may accumulate in the human thyroid significantly more than in other tissues [33,34,35]. In past years, in the absence of the over-screening phenomenon, heavy metal pollution has been indicated as a potential contributor to the increased TC incidence reported in volcanic and geothermal areas, such as the Hawaiian Islands and Iceland [36]. More recently, epidemiological studies conducted among recovery workers of World Trade Center (WTC-RRWs) exposed to potential carcinogens, including heavy metals, twenty years after the 11 September 2001 terrorist attacks showed that the incidence of TCs was similar at all stages, suggesting that the risk may also be increased independently of screening or surveillance bias [37]. One Korean study reported the results of a secondary analysis of a prospective cohort study among residents living near industrial complexes in South Korea, showing that urinary mercury concentration was positively associated with the risk of TC, suggesting the adverse effects of environmental metal pollution in the development of thyroid cancer [38]. In Italy, some studies have reported an increased incidence rate of TC in geographical areas characterized by the presence of natural or anthropometric environmental pollution sources. In Sicily, a marked increase in TC has been reported in the volcanic area of Mt. Etna; TC incidence was doubled relative to adjacent non-volcanic areas [39]. Other studies reported an excess risk of TC incidence near NPCS, suggesting a potential etiological role of residential exposure to endocrine disruptors in the development of TC [40].

Recently, another study reported a higher incidence of TC in Southern Tuscany, probably related to heavy metal contamination, mainly derived from mining sites spread throughout the province [18]. Similarities have been hypothesized with the Cyprus islet (the name Cyprus comes from the Latin word “cuprum,” which means copper, and the island is naturally rich in heavy metals), which is the first in Europe for TC incidence [41,42,43].

In our study, the observation that thyroid microcarcinomas of GMNR-exposed patients presented more extrathyroidal invasion compared to those who were not exposed strengthens the hypothesis that environmental factors could influence the phenotypic presentation of the neoplasm. Particularly, the higher incidence trends observed in advanced cancer (stages 3 and 4) seem to reduce the risk of surveillance bias. We found that a greater incidence rate of TC was observed in GMNR-exposed patients with a significant overall relative risk (RR) of 19.65 (*p* = 0.006), especially for male patients.

Patients born and living in municipalities in proximity to mines had a significant relative risk of TC of 1.46. A significant positive correlation was also found between the presence of NPCSs and TC incidence in male patients. We also evaluated the possible role of natural radioactivity as radon concentrations, although its effect on the thyroid is unknown [44,45]. In our series, we found that exposure to radon did not significantly increase the risk of diagnosing TC in both sexes.

The potential role of geothermal plants is controversial. In Iceland, a country rich with volcanoes and geothermal activity, the higher incidence of TC has remained stable from 1955 to 2020, excluding the hypothesis of over-screening [36,46]. A recent study describing the mortality of populations residing in geothermal areas of Tuscany during the period 2003–2012 reported an excess of mortality for all causes among males residing in the geothermal area of Southern Tuscany [47]. However, in our series, we did not find an increased risk of TC.

The strengths of this study are that it reported, for the first time, data on the incidence of TC in all ten provinces of the Tuscany region and correlates the TC incidence data with histological records of TC patients born and residents in Tuscany.

Some limitations of this study are the lack of information on the patient’s work activity and lifestyle factors (obesity, smoking), and we could not adjust our results for these several confounding factors, which were reported to be potential risk factors for TC. In addition, we lack information on social migration (some people could live in one province and work in another). However, some hypothesized risk factors for TC, such as obesity and iodine supply, do not appear to influence the incidence of TC in Tuscany. As reported by the first Italian National Observatory for Monitoring Iodine Prophylaxis (OSNAMI), Tuscany was a region with adequate iodine sufficiency [48]. In addition, the percentage of obese people (BMI ≥ 30) is significantly lower than the national level (8% vs. 10%) [49]. Moreover, we do not know the molecular profile of the TCs, although the observation that they are PTCs predominantly may indicate the involvement of carcinogenetic factors that influence specific molecular signalling that leads to PTC onset [50,51,52]. We have observed an increased prevalence of anaplastic carcinomas in the province of Prato, but the causes are unclear. A possible explanation is that the province of Prato has the highest average age at diagnosis compared to other TPs, and this possibility might suggest a potential relationship with the presence of a higher number of patients with long-standing goiter.

## 5. Conclusions

The overdiagnosis phenomenon is probably the main explanation for the increased incidence of thyroid cancer in the world, including Italy. However, we provided evidence that in some geographical areas, the presence of environmental pollution, especially that characterized by the release of heavy metals, might influence thyroid carcinogenesis and should be considered among the recognized risk factors for TC.

## Figures and Tables

**Table 1:** European standardized incidence rates (EU-standardized IR) (Per 100,000 age-standardized to the Italian population (2019) and 95% confidence intervals for thyroid cancer by sex (2013–2017) in the ten provinces in the entire Tuscany region and in Italy.

	Cases	EU-Standardized IR (95%)	SIR vs. Italy (95%)
Arezzo			
Males	75	8.4 (6.6–11.3)	0.890 (0.700–1.115)
Females	202	22.3 (19.3–25.7)	0.809 (0.701–0.929)
Pistoia			
Males	105	14.1 (11.5–17.1)	1.488 (1.217–1.801)
Females	233	29.4 (25.7–33.5)	1.094 (0.958–1.243)
Prato			
Males	68	10.8 (8.4–13.7)	1.093 (0.848–1.385)
Females	157	23.2 (19.7–33.5)	0.852 (0.724–0.997)
Firenze			
Males	316	12.3 (11–13.8)	1.303 (1.163–1.455)
Females	824	29.7 (27.7–31.9)	1.114 (1.040–1.193)
Massa-Carrara			
Males	68	12.7 (9.9–16.2)	1.420 (1.103–1.800)
Females	175	32.5 (27.8–37.8)	1.215 (1.041–1.409)
Lucca			
Males	168	16.5 (14.1–19.3)	1.777 (1.518–2.067)
Females	390	36.7 (33–40.6)	1.371 (1.238–1.514)
Pisa			
Males	167	15.5 (13.2–18)	1.620 (1.384–1.886)
Females	519	45.4 (41.5–49.5)	1.703 (1.559–1.855)
Livorno			
Males	136	15.5 (13–18.3)	1.664 (1.396–1.968)
Females	334	36 (32.1–40.1)	1.352 (1.211–1.505)
Siena			
Males	59	8.7 (6.6–11.3)	0.907 (0.690–1.169)
Females	172	23.9 (20.4–27.8)	0.872 (0.747–1.013)
Grosseto			
Males	88	14.9 (11.9–18.4)	1.625 (1.303–2.002)
Females	203	33.4 (28.9–38.5)	1.242 (1.077–1.425)
Tuscany			
Males	1250	13 (12.3–13.8)	1.379 (1.304–0.158)
Females	3209	31.6 (30.5–32.7)	1.177 (1.136–1.218)

**Table 2:** European standardized mortality rates (EU-standardized MR) (Per 100,000 age-standardized to the Italian Population (2019) and 95% confidence intervals for thyroid cancer by sex (2013–2017) in the ten provinces in the entire Tuscany region and in Italy.

	Cases	EU-Standardized MR (95%)	SIR vs. Italy (95%)
Arezzo			
Males	7	0.75 (0.28–1.22)	0.932 (0.375–1.920)
Females	10	0.81 (0.37–1.24)	1.130 (0.542–2.077)
Pistoia			
Males	12	1.53 (0.44–1.9)	1.113 (0.448–2.293)
Females	9	0.9 (0.4–1.41)	1.191 (0545–2.261)
Prato			
Males	<4		
Females	<4		
Total		0.29 (0.05–0.52)	
Firenze			
Males	14	0.56 (0.31–0.81)	0.278 (0.102–0.604)
Females	30	0.77 (0.53–1.01)	1.144 (0.772–1.633)
Massa-Carrara			
Males	<4		
Females	<4		
Total		0.67 (0.3–1.04)	
Lucca			
Males	11	1.01 (0.51–1.51)	1.306 (0.652–0.81)
Females	14	0.88 (0.48–1.27)	1.388 (0.759–2.329)
Pisa			
Males	5	0.52 (0.13–0.9)	1.307 (0.675–2.282)
Females	15	0.91 (0.52–1.31)	1.388 (0.777–2.289)
Livorno			
Males	6	0.6 (0.2–1)	1.922 (1.051–3.225)
Females	10	0.72 (0.34–1.1)	1.142 (0.547–2.099)
Siena			
Males	5	0.6 (0.15–1.04)	0.862 (0.280–2.012)
Females	8	0.8 (0.32–1.28)	1.144 (0.494–2.255)
Grosseto			
Males	7	1.17 (0.44–1.9)	1.036 (0.336–2.417)
Females	5	0.47 (0.12–0.81)	0.863 (0.280–2.013)
Tuscany			
Males	70	0.85 (0.68–1.02)	0.945 (0.732–1.200)
Females	111	0.77 (0.64–0.89)	1.187 (0.967–1.443)

**Table 3:** Clinicopathological features of thyroid cancer patients (n = 3210) according to the province of residence.

PARAMETERS	AR (n = 205)	FI (n = 893)	GR (n = 273)	LI (n = 332)	LU (n = 353)	MS (n = 193)	PI (n = 358)	PT (n = 232)	PO (n = 163)	SI (n = 208)	*p*
**Age at diagnosis (yrs)** Median	52	54	51	51	52	54	51	50	55	52.5	**0.001**
**Sex** Males females	49 (23.9) 156 (76.1)	234 (26.2) 659 (73.8)	76 (27.8) 197 (72.2)	107 (32.2) 225 (67.8)	103 (29.2) 250 (70.8)	55 (28.5) 138 (71.5)	80 (22.3) 278 (77.7)	66 (28.4) 166 (71.6)	53 (32.5) 110 (67.5)	52 (25) 156 (75)	0.1
**Histotypes: n (%)** Papillary Follicular Medullary Anaplastic	193 (94.2) 3 (1.5) 5 (2.4) 4 (1.9)	795 (89) 59 (6.6) 23 (2.6) 16 (1.8)	251 (91.9) 10 (3.7) 9 (3.3) 3 (1.1)	319 (96) 5 (1.5) 5 (1.5) 3 (1)	330 (93.5) 11 (3.1) 7 (2) 5 (1.4)	176 (91.2) 10 (5.2) 5 (2.6) 2 (1)	337 (94.1) 11 (3.1) 7 (2) 3 (0.8)	213 (91.8) 9 (3.9) 6 (2.6) 4 (1.7)	131 (80.3) 16 (9.8) 7 (4.3) 9 (5.5)	187 (89.9) 12 (5.8) 5 (2.4) 4 (1.9)	**<0.001**
**Size of tumour: (cm)** Median	1.1	1.1	1.3	1.1	1.1	1.2	1.3	1.2	1.0	1.5	0.1
Tumor extension: n (%) * T1a T1b T2 T3 T4 * (**TNM 8th Edition**)	96 (46.8) 66 (32.2) 21 (10.2) 17 (8.3) 5 (2.4)	431 (48.3) 233 (26.1) 126 (14.1) 84 (9.4) 19 (2.1)	94 (34.4) 82 (30.0) 39 (14.3) 54 (19.8) 4 (1.5)	125 (37.7) 97 (29.2) 33 (9.9) 70 (21.1) 7 (2.1)	143 (40.5) 106 (30.0) 54 (15.3) 45 (12.7) 5 (1.4)	68 (35.2) 60 (31.1) 24 (12.4) 36 (18.7) 5 (2.6)	129 (36.0) 112 (31.3) 57 (15.9) 55 (15.4) 5 (1.4)	92 (39.7) 69 (29.7) 41 (17.7) 23 (9.9) 7 (3.0)	78 (47.9) 30 (18.4) 23 (14.1) 21 (12.9) 11 (6.7)	82 (39.4) 64 (30.8) 43 (20.7) 14 (6.7) 5 (2.4)	**<0.001** **0.07** **0.03** **<0.001** **0.02**
**Lymph-node mts: n (%)** Yes No	36 (17.6) 169 (82.4)	108 (12.1) 785 (87.9)	41 (15) 232 (85)	55 (16.6) 277 (83.4)	31 (8.8) 322 (91.2)	31 (16.1) 162 (83.9)	44 (12.3) 314 (87.7)	46 (19.8) 186 (80.2)	26 (16) 137 (84)	35 (16.8) 173 (83.2)	**0.003**
**Bilaterality** Yes No	47 (23) 158 (77)	233 (26.2) 660 (73.8)	114 (41.8) 159 (58.2)	120 (36.1) 212 (63.9)	109 (31) 244 (69)	60 (31.1) 133 (68.9)	108 (30.2) 250 (69.8)	65 (28) 167 (72)	39 (23.9) 124 (76.1)	72 (34.8) 136 (65.2)	**<0.001**
**Multicentricity** Yes No	57 (27.8) 148 (72.2)	299 (33.5) 594 (66.5)	120 (44) 153 (56)	143 (43.1) 189 (56.9)	129 (36.5) 224 (63.5)	76 (39.4) 117 (60.6)	141 (39.4) 217 (60.6)	93 (40.1) 139 (59.9)	49 (30.1) 114 (69.9)	77 (37) 131 (63)	**0.001**

AR = Arezzo; FI = Firenze; GR = Grosseto; LI = Livorno; LU = Lucca; MS = Massa-Carrara; PI = Pisa; PT = Pistoia; PO = Prato; SI = Siena. The bold is to highlight statistically significant differences.

**Table 4:** Clinicopathological features of thyroid cancer patients, according to the residents in Tuscany municipalities, with presence (n = 385, exposed patients) and without (n = 2825, non-exposed patients) ascertained heavy metal contamination. The bold is to highlight statistically significant differences.

PARAMETERS	No Exposed (n = 2825)	Exposed (n = 385)	*p*
**Age at diagnosis (yrs)** Median	53	52	0.1
**Sex: n (%)** Males females	763 (27) 2062 (73)	112 (29) 273 (71)	0.4
**Histotypes: n (%)** Papillary Follicular Medullary Anaplastic*	2571 (92.4) 139 (5.0) 72 (2.6) 43 (1.5)	365 (95.5) 11 (2.9) 6 (1.6) 3	0.03 0.1 0.3
**Size of tumour: (cm)** Median	1.2	1.2	0.8
Tumor extension: n (%) * T1a T1b T2 T3 T4 * (**TNM 8th Edition**)	1201 (42.5) 796 (28.2) 426 (15.1) 366 (13.0) 36 (1.3)	137 (35.6) 125 (32.5) 125 (32.5) 73 (19.0) 6 (1.6)	0.02 0.02 0.1 <0.001 1.0
**Lymph-node mts: n (%)** Yes No	396 (14) 2429 (86)	57 (14.8) 328 (85.2)	0.7
**Bilaterality** Yes No	819 (29) 2006 (71)	148 (38.4) 237 (61.6)	<0.001
**Multicentricity** Yes No	1087 (38.5) 1738 (61.5)	181 (47) 204 (53)	0.002

**Table 5:** Incidence rate (Per 100,000 inhabitants and 95% confidence intervals) and relative risk of thyroid cancer in relation to the presence of heavy metal contamination sources.

Population	Incidence Rate per 100,000 Inhabitants (95% CI)	Risk Ratio (95% CI)	*p*-Value
GMNR-non-exposed			
overall	16.90 (16.28–17.54)	NA	NA
males	9.49 (8.83–10.19)	NA	NA
females	23.77 (22.76–24.82)	NA	NA
GMNR-exposed			
Overall	19.65 (17.73–21.71)	1.16 (1.04–1.29)	**0.006**
males	11.94 (9.83–14.36)	1.26 (1.03–1.53)	**0.03**
females	26.73 (23.65–30.09)	1.12 (0.99–1.28)	0.07
mine,			
overall	24.67 (19.62–30.62)	1.46 (1.17–1.82)	**0.001**
males	12.59 (7.69–19.45)	1.33 (0.85–2.07)	0.19
females	35.72 (27.39–45.79)	1.50 (1.17–1.93)	**0.003**
NPCS			
overall	18.99 (16.77–21.43)	1.12 (0.99–1.27)	0.07
males	12.79 (10.22–15.82)	1.35 (1.08–1.69)	**0.01**
females	24.67 (21.19–28.56)	1.04 (0.89–1.21)	0.63
radon			
overall	18.94 (13.65–25.59)	1.12 (0.83–1.52)	0.46
males	8.39 (3.84–15.92)	0.88 (0.46–1.71)	0.87
females	28.82 (19.84–40.47)	1.21 (0.86–1.71)	0.29
geothermal power plant			
overall	16.01 (10.72–22.99)	0.95 (0.66–1.37)	0.86
males	5.71 (1.85–13.33)	0.60 (0.25–1.45)	0.38
females	25.64 (16.43–38.15)	1.08 (0.72–1.61)	0.67

## Data Availability

The original contributions presented in this study are included in the article. Further inquiries can be directed at the corresponding author.

## Supplementary Materials

The following supporting information can be downloaded at: https://www.mdpi.com/article/10.3390/cancers17050717/s1, Table S1: List of municipalities exposed to geothermal power plants, mines, National Priority Contaminated Sites (NPCs) and radon.
