# Supplementary material for: Epidemiology of Thyroid Cancer in Tuscany (Central Italy) 2013–2017: Not Just Overdiagnosis
Source: Cancers (Basel). 2025 Feb 20;17(5):717. doi: 10.3390/cancers17050717 (PMC11899145; doi:10.3390/cancers17050717)
Supplement: Supplementary file 1 [file cancers-17-00717-s001.zip › cancers-3465350-supplementary.docx]

**Supplementary Table 1.** List of municipalities exposed to geothermal power plants, mines, National Priority Contaminated Sites (NPCs) and radon.

| **Municipality** | **Province** | **Exposed to** | **5-year population*** | **TC Cases*** | **Proportion ≥55 years** | **Male/ female ratio** | **Km from nearest provincial capital** |
| --- | --- | --- | --- | --- | --- | --- | --- |
| Abbadia San Salvatore | SI | geothermal power plant, radon | 32452 | 4 | 0.466 | 0.884 | 47.4 |
| Arcidosso | GR | geothermal power plant, radon | 21754 | 4 | 0.405 | 0.948 | 36.8 |
| Campiglia marittima | LI | mine | 66137 | 25 | 0.423 | 0.924 | 51.4 |
| Castel del Piano | GR | geothermal power plant, radon | 23405 | 6 | 0.413 | 0.925 | 37.5 |
| Castell’Azzara | GR | geothermal power plant, mine | 7567 | 1 | 0.569 | 0.910 | 48.3 |
| Castelnuovo Val di Cecina | PI | geothermal power plant | 11280 | 4 | 0.460 | 0.941 | 37 |
| Chiusdino | SI | geothermal power plant | 9562 | 0 | 0.407 | 0.949 | 27.3 |
| Gavorrano | GR | mine | 43512 | 8 | 0.413 | 0.938 | 23.5 |
| Isola Giglio | GR | radon | 7136 | 1 | 0.455 | 1.130 | 48.3 |
| Livorno | LI | NPC | 797711 | 134 | 0.404 | 0.913 | 0 |
| Marciana | LI | radon | 10938 | 0 | 0.465 | 0.937 | 76.7 |
| Massa | MS | NPC | 346551 | 75 | 0.404 | 0.927 | 0 |
| Massa marittima | GR | mine | 42589 | 7 | 0.454 | 0.901 | 35.5 |
| Montecatini Val di Cecina | PI | radon | 8907 | 2 | 0.467 | 0.979 | 38.7 |
| Monterotondo marittimo | GR | geothermal power plant | 6829 | 1 | 0.414 | 1.066 | 42.9 |
| Montieri | GR | mine | 6138 | 0 | 0.443 | 1.031 | 33.1 |
| Orbetello | GR | NPC | 74523 | 16 | 0.440 | 0.897 | 37.9 |
| Piancastagnaio | SI | geothermal power plant, radon | 21137 | 4 | 0.427 | 0.923 | 47.9 |
| Pietrasanta | LU | mine | 120124 | 29 | 0.434 | 0.889 | 11.8 |
| Piombino | LI | NPC | 171355 | 39 | 0.444 | 0.913 | 51.1 |
| Pitigliano | GR | radon | 19310 | 2 | 0.459 | 0.898 | 48.7 |
| Pomarance | PI | geothermal power plant | 29423 | 3 | 0.486 | 0.961 | 37.3 |
| Radicondoli | SI | geothermal power plant | 4631 | 1 | 0.444 | 0.967 | 24.3 |
| Roccastrada | GR | mine, radon | 46314 | 12 | 0.441 | 0.951 | 27 |
| Santa Fiora | GR | geothermal power plant, radon | 13118 | 1 | 0.460 | 0.953 | 41 |
| Sorano | GR | radon | 17336 | 6 | 0.510 | 0.960 | 50.5 |

TC, thyroid cancer; SI, Siena, GR, Grosseto; LI, Livorno; PI, Pisa; MS, Massa-Carrara; LU, Lucca; NPC, National Priority Contaminated Site.

* aggregate number from 2013 to 2017
